# The clinical impact of pain neuroscience continuing education on physical therapy outcomes for patients with low back and neck pain

**DOI:** 10.1371/journal.pone.0267157

**Published:** 2022-04-28

**Authors:** Adriaan Louw, Emilio J. Puentedura, Thomas R. Denninger, Adam D. Lutz, Terry Cox, Kory Zimney, Merrill R. Landers

**Affiliations:** 1 Evidence in Motion, Story City, Iowa, United States of America; 2 Department of Physical Therapy, Robbins College of Health and Human Sciences, Baylor University, Waco, Texas, United States of America; 3 ATI Physical Therapy, Greenville, South Carolina, United States of America; 4 Department of Physical Therapy, Southwest Baptist University, Bolivar, Missouri, United States of America; 5 Department of Physical Therapy, School of Health Sciences, University of South Dakota, Vermillion, South Dakota, United States of America; 6 Department of Physical Therapy, School of Integrated Health Sciences, University of Nevada Las Vegas, Las Vegas, Nevada, United States of America; Universiti Sains Malaysia, MALAYSIA

## Abstract

**Objectives:**

Research suggests that attendance by physical therapists at continuing education (CE) targeting the management of low back pain (LBP) and neck pain does not result in positive impacts on clinical outcomes. The aim of this study was to determine if therapists attending a self-paced 3-hour online Pain Neuroscience Education (PNE) program was associated with any observed changes to patient outcomes and also clinical practice.

**Methods:**

Participants were 25 different physical therapists who treated 3,705 patients with low back pain (LBP) or neck pain before and after they had completed an online PNE CE course. Change in outcomes measures of pain and disability at discharge were compared for the patients treated before and after the therapist training. Clinical practice patterns of the therapists, including total treatment visits, duration of care, total units billed, average units billed per visit, percentage of ‘active’ billing units and percentage of ‘active and manual’ billing units, were also compared for the patient care episodes before and after the therapist training.

**Results:**

There was no significant difference for change in pain scores at discharge for patients treated after therapist CE training compared to those treated before regardless of the condition (LBP or neck pain). However, patients with LBP who were treated after therapist CE training did report greater improvement in their disability scores. Also after CE training, for each episode of care, therapists tended to use less total visits, billed fewer units per visit, and billed a greater percentage of more ‘active’ and ‘active and manual’ billing units.

**Discussion:**

Attending an online 3-hour CE course on PNE resulted in improved disability scores for patients with LBP, but not for those with neck pain. Changes in clinical behavior by the therapists included using less visits, billing fewer total units, and shifting to more active and manual therapy interventions. Further prospective studies with control groups should investigate the effect of therapist CE on patient outcomes and clinical practice.

## Introduction

The ultimate aim of clinical research is to better the lives of the end-user, the patient [1]. With new discoveries, a systematic scientific process takes an initial concept through various stages of development and validation, until its clinical impact is measured. Early exploration often includes a case study, the development of case series or clinical prediction rules, with no control groups [2,3]. Once a new concept has been initially tested, it undergoes a more robust series of tests, including control groups, blinding of participants and scientists, ultimately leading to high-level randomized clinical trials, systematic reviews and meta-analyses speaking to the efficacy of a certain therapeutic intervention or approach [4–6]. One final element, however, is often missing in clinical research and that is implementation science. Implementation science is the study of the uptake of evidence-based practice and research into clinical practice by practitioners and policymakers [7]. In this final stage, the intent is to see if this new intervention or approach is disseminated into clinical practice and impacts patients at scale, when compared to previous approaches.

Once a new intervention or approach is developed and validated, clinical researchers have to find a way to disseminate their work to front-line clinicians, with the intent to impact the patient. To do so, post-professional continuing education (CE) is often used [8]. In most professions, post-professional CE is required to maintain licensure and address issues such as competency, ethics, safety and compliance. Importantly, it is also an opportunity to impart the latest evidence from research to prevent clinical obsolescence, to convey contemporary professional trends, and to promote adherence to established clinical guidelines [8–11]. To date, however, post-professional CE has shown limited efficacy in altering treatment behaviors in healthcare providers. As an example, studies have failed to demonstrate that PT CE targeting LBP and neck pain can positively impact clinical outcomes [12–16].

In musculoskeletal care, an educational approach known as pain neuroscience education (PNE) has emerged as a means to teach people more about the underlying biology and physiology of their pain experience [17–20]. The primary goal of PNE is to increase a person’s knowledge about pain—how pain works biologically and physically—as a means to improve their pain experience [20–22]. Current best-evidence shows strong support for PNE positively influencing self-reported pain ratings, pain knowledge, disability, pain catastrophizing, fear-avoidance, beliefs regarding pain, physical movement and healthcare utilization and costs [18–20,23,24]. Furthermore, studies have shown that healthcare providers who have been taught PNE demonstrate increased knowledge of pain, are positively impacted in their attitudes and beliefs regarding chronic pain, and they become more empathetic and compassionate towards people with chronic pain [25–29]. In regards to clinical impact, a one-year follow-up, self-reported study showed that 270 healthcare providers who attended a single, 3.5-hour PNE lecture reported large positive impacts in daily clinical practice based on the educational course [29]. The results, however, were self-reported, so there was no validation that changes to clinical practice had actually occurred.

The aims of this study were two-fold. First, to determine if therapists attending a self-paced 3-hour online PNE program was associated with any observed changes to their clinical practice. To achieve this, we compared their clinical outcomes for patients with LBP and neck pain prior to the PNE with their clinical outcomes for patients with LBP and neck pain after completing the PNE program. Our second aim was to determine if those therapists exhibited more efficient treatment, employed more active treatment approaches, and displayed more cost-effective billing/care patterns after completing the 3-hour online PNE program.

## Methods

### Study design

This study was a retrospective cohort study using de-identified episodic patient data provided by a large national outpatient PT provider (BLINDED). All data (patient and therapist) was fully anonymized prior to access and therefore, the Institutional Review Board obtained from (BLINDED) University and ethics committee from (BLINDED) PT waived the requirement for informed consent. Only patient episodes for whom the primary treating physical therapist completed a 3-hour online PNE course were examined. Patient reported outcomes and other data derived from these patient episodes were compared prior to- and post- the 3-hour online PNE continuing education course.

### Participants

A convenience sample yielded 3,705 completed patient cases of LBP and neck pain (Table 1) who were treated by 25 different physical therapists who had completed the online PNE course (Table 2). There were 2,216 completed patient care episodes treated within the 6-month period prior to the therapist starting their PNE course. A 2-week washout period was instituted for patient care cases after each therapist’s PNE training, and then 1,489 completed care episodes treated within the 6-month period after the PNE training. To examine for differences in pre- and post- PNE course outcomes, patient cases were included if the patients were discharged prior to the therapist starting the course or evaluated after the course was completed. Patient cases where the episode of care spanned the beginning and/or completion of the PNE course were excluded.

**Table 1:** Patient descriptive data of the 3,705 completed cases broken up into back or neck pain represented in means with standard deviations and proportions for categorical variables.

			Back (n = 2464)	Before (n = 1457)	After (n = 1007)	Neck (n = 1241)	Before (n = 759)	After (n = 482)
**Patient characteristics**	Sex	Females	n = 1436 (58.3%)	n = 847 (58.1%)	n = 589 (58.5%)	n = 776 (62.5%)	n = 479 (63.1%)	n = 297 (61.6%)
		Males	n = 1028 (41.7%)	n = 610 (41.9%)	n = 418 (41.5%)	n = 465 (37.5%)	n = 280 (36.9%)	n = 185 (38.4%)
	Age		53.7 ± 17.8	52.8 ± 17.6	54.9 ± 17.9	51.1 ± 16.2	51.0 ± 16.0	51.3 ± 16.6
	Body Mass Index		29.7 ± 6.6	29.8 ± 6.6	29.5 ± 6.5	28.3 ± 6.2	28.4 ± 6.2	28.1 ± 6.3
	Payor status	Commercial	n = 1067 (43.3%)	n = 596 (40.9%)	n = 471 (46.8%)	n = 463 (37.3%)	n = 275 (36.2%)	n = 188 (39.0%)
		Medicare	n = 550 (22.3%)	n = 320 (22.0%)	n = 230 (22.8%)	n = 169 (13.6%)	n = 100 (13.2%)	n = 69 (14.3%)
		Medicaid	n = 397 (16.1%)	n = 277 (19.0%)	n = 120 (11.9%)	n = 187 (15.1%)	n = 124 (16.3%)	n = 63 (13.1%)
		Workers’ comp	n = 237 (9.6%)	n = 137 (9.4%)	n = 100 (9.9%)	n = 95 (7.7%)	n = 57 (7.5%)	n = 38 (7.9%)
		Auto/Personal injury	n = 191 (7.8%)	n = 116 (8.0%)	n = 75 (7.4%)	n = 312 (25.1%)	n = 195 (25.7%)	n = 117 (24.3%)
	Duration of symptoms	< 6 months	n = 1775 (72.0%)	n = 1021 (70.1%)	n = 754 (74.9%)	n = 952 (76.7%)	n = 566 (74.6%)	n = 386 (80.1%)
		> 6 months	n = 689 (28.0%)	n = 436 (29.9%)	n = 253 (25.1%)	n = 289 (23.3%)	n = 193 (25.4%)	n = 96 (19.9%)
	Total comorbidities		3.2 ± 2.9	3.1 ± 2.7	3.2 ± 3.1	3.0 ± 2.8	3.1 ± 2.8	2.9 ± 2.7
**Initial presentation**	Disability		38.3 ± 18.2	38.9 ± 18.5	37.4 ± 17.7	38.9 ± 18.7	39.8 ± 18.7	37.4 ± 18.4
	Pain at rest		3.5 ± 2.1	3.7 ± 2.1	3.3 ± 2.0	4.0 ± 2.5	4.1 ± 2.5	3.8 ± 2.6
	Pain during activity		7.4 ± 2.1	7.5 ± 2.1	7.3 ± 2.1	7.3 ± 2.1	7.3 ± 2.1	7.2 ± 2.1
	Mental component score		39.3 ± 7.4	39.3 ± 7.4	39.4 ± 7.3	39.4 ± 7.4	39.2 ± 7.6	39.7 ± 7.1
	Physical component score		37.5 ± 6.4	37.4 ± 6.3	37.5 ± 6.6	37.6 ± 6.4	37.4 ± 6.4	37.8 ± 6.4
**Data from episode of care**	Number of patient visits		14.1 ± 7.5	14.3 ± 7.3	13.9 ± 7.8	14.9 ± 8.3	15.3 ± 8.2	14.2 ± 8.5
	Duration of care (days)		54.9 ± 37.2	58.2 ± 39.8	52.3 ± 35.0	55.9 ± 38.6	58.3 ± 42.0	53.6 ± 34.7
	Disability change (pre–post)		9.5 ± 15.7	8.8 ± 15.7	8.8 ± 15.7	11.0 ± 15.7	11.6 ± 16.1	10.1 ± 14.9
	Residual disability change (pre–post)		-.064 ± 14.0	-.727 ± 14.0	.896 ± 13.9	-.471 ± 13.8	.013 ± 14.0	-1.23 ± 13.6

**Table 2:** Physical therapy descriptive data represented in means with standard deviations and proportions for categorical variables.

	n = 25
Gender	Females = 14 (56.0%) Males = 11 (44.0%)
Year of experience	9.2 ± 9.0 (range: 2, 39)
American Board of Physical Therapy Specialties–clinical specialist	No = 19 Yes = 6
American Board of Physical Therapy Residency and Fellowship Education residency-trained	No = 20 Yes = 5

### PNE program

Therapists working for the national PT group had been given access to online, self-directed CE content, including PNE. The content of PNE is well-documented and consistent with other studies [30–32]. A 3-hour presentation focusing on peripheral sensitization, central sensitization, biopsychosocial factors associated with pain, threat appraisal of the brain, nociception, stress and endocrine responses to pain, and various therapeutic endogenous strategies to ease pain were used [30,31,33]. Images, metaphors and examples were used along with the educational content [34,35].

### Patient outcome measures

For Aim 1, the following 4 outcomes were analyzed for each of the completed patient cases before and after the PNE training program: 1) change in pain at rest; 2) change in pain with activity; 3) change in disability; and, 4) residual disability.

Change in pain at rest and with activity were analyzed using the numeric pain rating scale (NPRS). The NPRS is an 11-point numeric scale anchored with ‘0’ representing one pain extreme (no pain) and ‘10’ respecting the other pain extreme (worst pain imaginable) [36]. Change in pain at rest and with activity was calculated by subtracting the NPRS score at discharge from the score at initial evaluation for each patient case. The minimally clinically important difference (MCID) for the NPRS for LBP and neck pain have been reported to range between 1.5 and 2.2 points respectively [36–38], and it is generally accepted that a 2-point change on the NPRS represents clinically meaningful change [39]. For this study, a change of ≥ 2 points was chosen as meeting the MCID for the NPRS.

Disability was ascertained using the Neck Disability Index (NDI) [40] for cases with neck pain cases and the Oswestry Disability Index (ODI) for cases with LBP [41]. Both the NDI and the ODI are condition-specific outcome measures comprising 10-item questionnaires which are scored from 0 to 5, leading to a disability score with a maximum of 50. In this study, the patient-reported score was calculated as a percentage of the total possible points. The MCID for the ODI has been reported to be approximately 6 points (12% change) [42], whereas for the NDI it can range from 3.5 to 8.5 points (7–19% change) depending upon the neck condition [43–46].

Change in disability was calculated by subtracting the percentage disability at discharge from the percentage disability at initial evaluation for each patient case. For this study, a change of ≥ 12% was chosen as meeting the MCID for the ODI [42], and a change of ≥ 15% for the NDI [43,44].

Residual disability was based off recent research which defined it as actual patient reported outcome change (minus) risk adjusted predicted patient reported outcome change [47]. A positive residual value indicated change in disability was greater than prediction, while a negative residual value indicated change in disability was less than prediction [48].

For Aim 2, the following 6 outcomes were analyzed for each of the completed patient cases before and after the PNE training program: 1) number of total treatment visits; 2) total duration of care in days; 3) number of total units billed for the entire case; 4) average number of units billed per visit; 5) percentage of “active” billing units relative to total units billed and 6) percentage of “active and manual” billing units relative to total billing units. “Active” billing units were considered therapeutic exercise (CPT code 97110), therapeutic activity (CPT code 97530), neuromuscular reeducation (CPT code 97112), and gait training (CPT code 97116). “Active and Manual” billing units were considered any active billing units as above plus manual therapy (CPT code 97140).

### Data analysis

Data were analyzed using SPSS version 28.0 (IBM SPSS Statistics for Windows, Armonk, New York, USA: IBM Corp) at α = 0.05. Cases with missing data were removed from the analysis. For Aim 1, a 2 (time: before and after PNE) X 2 (region: back and neck) ANCOVA was conducted for each of the four outcomes: pain at rest, pain with activity, disability, and residual disability. The following physical therapist characteristics were included as covariates in the analysis: years of experience, gender, training (yes or no on American Board of Physical Therapy Specialties (ABPTS) certification and/or residency). The following patient characteristics were included as covariates in the analysis: gender, body mass index, median income determined by zip code, payor status (yes or no for each of the following: Workers’ compensation, personal injury, Medicare, Medicaid, and Commercial), age, number of total visits, number of total comorbidities. Because collinearity was found among training groups (certification and residency), those separate categories were combined into one variable. Gender concordance of the therapist and patient was not statistically correlated with any outcome and was, subsequently, omitted from the ANCOVA. Likewise, initial mental and physical component scores were not correlated with any outcome, so both were removed. Collinearity was observed between duration of care and total number of PT visits, so only total number of visits was retained.

For Aim 2, a 2 (time: before and after PNE) X 2 (region: back and neck) ANCOVA was conducted for each of the 6 outcomes: number of total treatment visits, total duration of care in days, number of total units billed for the whole case, average number of units billed per visit, percentage of “active” billing units relative to total units, and percentage of “active and manual” billing units relative to total billing units. The covariates entered in the Aim 2 analyses were the same as Aim 1.

## Results

### Pain change at rest and with activity

With covariates added, there was no interaction between time and region, F(1,2366) = .003, p = .958 *at rest* and F(1,2629) = 2.169, p = .140 *with activity* (Table 3). Likewise, there was not a statistically significant main effect for either region (p = .060) or time (p = .709) *at rest* (p = .250) or *with activity* (p = .800).

**Table 3:** Pain change (NPRS initial–NPRS discharge) for before and after PNE and by body region.

	Pain at rest	Mean	SD	n	Pain with activity	Mean	SD	n
Before PNE	Back	1.72	2.24	978	Back	2.72	2.76	1050
	Neck	2.10	2.20	498	Neck	3.12	2.70	549
	Total	1.84	2.23	1476	Total	2.86	2.75	1599
After PNE	Back	1.77	2.21	602	Back	2.97	2.87	689
	Neck	2.12	2.30	289	Neck	3.00	2.77	342
	Total	1.88	2.25	891	Total	2.98	2.84	1031
Total	Back	1.74	2.23	1580	Back	2.82	2.81	1739
	Neck	2.11	2.24	787	Neck	3.07	2.72	891
	Total	1.86	2.24	2367	Total	2.90	2.78	2630

### Disability (NDI/ODI)

With covariates added, there was a statistically significant interaction between time and region, F(1,3308) = 9.672, p = .002 (Table 4). Post hoc analyses, with a Bonferroni correct alpha of .025 (comparison of pre- and post- for both regions) revealed that patients with LBP had greater improvement in disability scores with therapists after the PNE course than those treated by the same therapists before the PNE course (p = .004). There was no statistically significant change in patients with neck pain (p = .080).

### Residual disability

Results for residual disability paralleled disability. There was a statistically significant interaction between time and region, F(1,3308) = 8.718, p = .003 (Table 4). Post hoc analyses, with a Bonferroni correct alpha of .025 (comparison of pre- and post- for both regions) revealed that patients with LBP had greater residual disability improvement with therapists after the PNE course than those treated by the same therapists before the PNE course (p = .005). There was no statistically significant change in patients with neck pain (p = .122).

**Table 4:** Change in disability and residual disability for before and after PNE and by body region.

	Disability	Mean	SD	n	Residual Disability	Mean	SD	n
Before PNE	Back	8.7*	15.83	1307	Back	-0.72*	14.06	1307
	Neck	11.5	16.15	698	Neck	-0.05	14.13	698
	Total	9.6	16.00	2005	Total	-0.48	14.08	2005
After PNE	Back	11.0*	15.55	878	Back	1.29*	13.71	878
	Neck	10.0	14.53	426	Neck	-1.09	12.91	426
	Total	10.7	15.23	1304	Total	0.51	13.50	1304
Total	Back	9.6	15.76	2185	Back	0.09	13.95	2185
	Neck	10.9	15.57	1124	Neck	-0.44	13.68	1124
	Total	10.0	15.70	3309	Total	-0.09	13.86	3309

* Indicates statistically significant difference.

### Number of visits

With covariates added, there was no interaction between time and region, F(1,3308) = 1.297, p = .255 (Table 5). There was not a statistically significant main effect for region (p = .656) but there was for time (p = .017) with fewer visits reported after PNE than before PNE, regardless of body region.

### Duration of care

With covariates added, there was no interaction between time and region, F(1,2388) = .203, p = .652 (Table 5). Likewise, there was not a statistically significant main effect for either region (p = .263) or time (p = .069).

### Number of total units billed for the whole case

With covariates added, there was no interaction between time and region, F(1,2387) = 1.310, p = .252 (Table 6). There was not a statistically significant main effect for region (p = .893) but there was for time (p = .011) which indicates fewer billing codes used after PNE regardless of body region.

**Table 5:** Change in number of visits and duration of care (in days) for before and after PNE and by body region.

	Number of visits	Mean	SD	n	Duration of care	Mean	SD	n
Before PNE	Back	14.1	7.08	1307	Back	57.9	39.59	708
	Neck	15.2	8.25	698	Neck	57.4	36.32	403
	Total	14.5*	7.52	2005	Total	57.7	38.42	1111
After PNE	Back	13.8	7.89	878	Back	51.9	35.44	862
	Neck	14.2	8.62	426	Neck	53.2	35.68	417
	Total	13.9*	8.14	1304	Total	52.3	35.51	1279
Total	Back	14.0	7.42	2185	Back	54.6	37.47	1570
	Neck	14.8	8.40	1124	Neck	55.3	36.03	820
	Total	14.3	7.77	3309	Total	57.9	39.59	708

* Indicates statistically significant difference.

**Table 6:** Change in total units billed for whole case, and average number of billing units per visit for before and after PNE and by body region.

	Total units billed	Mean	SD	n	Average number of billing units per visit	Mean	SD	n
Before PNE	Back	64.5	45.56	708	Back	4.3	1.48	708
	Neck	71.7	51.80	403	Neck	4.5	1.24	403
	Total	67.1*	48.02	1111	Total	4.4	1.40	1111
After PNE	Back	61.3	47.17	860	Back	4.5	3.19	860
	Neck	65.2	51.74	417	Neck	4.7	3.20	417
	Total	62.6*	48.72	1277	Total	4.5	3.20	1277
Total	Back	62.7	46.46	1568	Back	4.4	2.56	1568
	Neck	68.4	51.84	820	Neck	4.6	2.45	820
	Total	64.7	48.44	2388	Total	4.5	2.53	2388

* Indicates statistically significant difference.

### Average number of billing units per visi

With covariates added, there was no interaction between time and region, F(1,2387) = .162, p = .687 (Table 6). Likewise, there was not a statistically significant main effect for either region (p = .954) or time (p = .146).

### Percentage active billing units

With covariates added, there was no interaction between time and region, F(1,2387) = 2.169, p = .140 (Table 7). However, there was a statistically significant main effect for region (p < .001), indicating that patients with LBP received a higher percentage of active treatment than patients with neck pain regardless of time. Additionally, there was a statistically significant main effect for time (p < .001), indicating that patients received a higher percentage of active treatment after PNE regardless of body region.

**Table 7:** Change in percentage of active billing units, and percentage of active and manual billing units per visit for before and after PNE and by body region.

	Percentage active billing units	Mean	SD	n	Percentage active and manual billing units	Mean	SD	n
Before PNE	Back	.526	.267	708	Back	.679	.283	708
	Neck	.419	.248	403	Neck	.624	.284	403
	Total	.487*	.265	1111	Total	.659*	.285	1111
After PNE	Back	.592	.247	860	Back	.758	.268	860
	Neck	.481	.232	417	Neck	.693	.257	417
	Total	.556*	.248	1277	Total	.737*	.266	1277
Total	Back	.562*	.258	1568	Back	.722*	.278	1568
	Neck	.451*	.241	820	Neck	.659*	.273	820
	Total	.524	.258	2388	Total	.701	.278	2388

* Indicates statistically significant difference.

### Percentage active and manual billing units

With covariates added, there was no interaction between time and region, F(1,2387) = .014, p = .905 (Table 7). However, there was a statistically significant main effect for region (p = .001), indicating that patients with LBP received a higher percentage of active and manual treatment than patients with neck pain regardless of time. Additionally, there was a statistically significant main effect for time (p < .001), indicating that patients received a higher percentage of active and manual treatment after PNE regardless of body region.

## Discussion

Results from this study show that there was no significant change in pain scores for patients with LBP or neck pain when they were treated by physical therapists after they had attended an online, self-directed CE course on PNE. The total mean change in pain *at rest* was 1.9 points and the total mean change in pain *with activity* was 2.9, indicating that patients reported decreased pain regardless of region (LBP and neck pain). While there was a statistically significant change in disability scores and residual disability for patients with LBP pre- and post-CE, the mean change scores did not meet our 12% threshold to be considered clinically meaningful. The PNE course utilized in this retrospective study was online, self-directed, and only 3 hours in duration. It may not have been of sufficient rigor and duration to make any difference in outcomes, however; these findings are similar to several studies that examined the effect of clinicians attending CE on patient reported outcomes for LBP and neck pain. Brennan et al. [12] examined NDI change scores for 1,365 patients with neck pain pre- and post-attendance by 34 physical therapists at a 2-day CE course and found no improvement. Chipchase et al. [14] compared NDI change scores for patients with neck pain who were treated by physical therapists who attended a 2-day CE course versus a 2-day CE course with a 5-hour follow-up session one month after training. They found no significant differences between the groups for patient outcomes [14]. In a more recent cluster randomized controlled trial, 12 groups of Finnish physical therapists and physicians were given 3–7 days of training focusing on the biopsychosocial management of LBP and compared with 15 control groups that did not receive training [49]. They found no significant difference in ODI between the groups [49].

This study did find that fewer number of visits were used by therapists after PNE than before PNE, regardless of body region treated. However, the mean difference was 0.6 visits and there was no difference in the total duration of care in days. Perhaps associated with this, was the finding that the total number of units billed for each case was less after PNE regardless of body region treated. The mean difference was 4.5 units, and this was also the average number of billing codes per visit, which did not show any change. As the cost of the average billing unit charged by the national outpatient PT provider was $30, this represents a cost savings of $135 per patient. There were 1,277 patients seen by the therapists after PNE training, which would indicate a total of $172,395 saved.

Finally, the data showed that while therapists billed a greater percentage of active billing units for cases of LBP regardless of time (56.2% vs 45.1%), they also billed a greater percentage of active billing units after the PNE training regardless of body region treated (55.6% vs 48.7%). A shift from passive interventions (billing codes) to more active treatments suggests a greater focus on patients’ health locus of control and self-efficacy which have been shown to moderate rehabilitation outcomes in people with LBP [50,51]. This was information covered in the PNE program and it is also in line with current clinical practice guidelines for LBP [6] and neck pain [52]. Similar results were found for the percentage of active and manual billing units. Therapists billed a greater percentage of active and manual billing units for cases of LBP regardless of time (72.2% vs 65.9%), and they also billed a greater percentage of active and manual billing units after the PNE training regardless of body region treated (73.7% vs 65.9%). The increased provision of active and manual treatments indicates that therapists were providing more ‘hands-on’ treatments with the active interventions following their PNE training, and this may be seen as a greater emphasis on high-value interventions and meeting the patients’ needs. The most recent clinical practice guidelines for interventions for the management of LBP gave a grade of A (strong evidence) for manual therapy (thrust and non-thrust joint mobilization) in the management of both acute and chronic LBP [53].

### Limitations

The study contains various limitations. First, there was no control group to compare the PNE group to, which limits the causal inference of this study. Secondly, this study was limited to the use of a 3-hour self-paced online PNE CE course, and there was no opportunity to explore of the optimal dosage of PNE CE. Future studies should explore non-PNE interventions and possibly varying durations and delivery messages for optimal PNE CE training. Finally, the data made available to us for this sample of therapists and treatments was from one large PT group which may well reflect the organization’s philosophy, in part, to treating LBP and neck pain and care should be taken to extrapolate these to all PTs treating LBP and neck pain. Future studies should explore therapists that are representative of various clinical settings, organizations, regions, and backgrounds.

## Conclusion

While attending an online 3-hour CE course on PNE was not associated with improved patient reported pain and disability outcomes for cases of neck pain, it was associated with improved disability outcomes for cases of LBP. For both LBP and neck pain, it did appear to result in some changes in the clinical behavior of the therapists. Therapists used less PT visits, billed fewer total units, and shifted their billing to more active and manual therapy interventions following their training. We recommend prospective studies with control groups to further explore the effect of CE on patient outcomes and clinical practice.

## Supporting information

S1 File (SAV)
